# Binary Effects of Gynostemma Gold Nanoparticles on Obesity and Inflammation via Downregulation of PPARγ/CEPBα and TNF-α Gene Expression

**DOI:** 10.3390/molecules27092795

**Published:** 2022-04-27

**Authors:** Reshmi Akter, Li Ling, Esrat Jahan Rupa, Jin KyuPark, Ramya Mathiyalagan, Jinnatun Nahar, Lee Jong Won, Kim Do Hyun, Mohanapriya Murugesan, Deok Chun Yang, Se Chan Kang, Gi-Young Kwak

**Affiliations:** 1 Graduate School of Biotechnology, College of Life Sciences, Kyung Hee University, Yongin-si 17104, Korea; reshmiakterbph57@gmail.com (R.A.); eshratrupa91@gmail.com (E.J.R.); pjinkyu53@gmail.com (J.K.); ramyabinfo@gmail.com (R.M.); jinnatunnaharbph@gmail.com (J.N.); priyabioinfo@khu.ac.kr (M.M.); dcyang@khu.ac.kr (D.C.Y.); 2 Department of Oriental Medicinal Biotechnology, College of Life Sciences, Kyung Hee University, Yongin-si 17104, Korea; aqling@naver.com; 3 Korea Hongsam Won, CO., 38, Ganghwa-daero 833beon-gil, Songhae-myeon, Ganghwa-gun, Incheon 23019, Korea; bbanvov@naver.com (L.J.W.); kkh880208@naver.com (K.D.H.)

**Keywords:** obesity, inflammation, *Gynostemma pentaphyllum*, gold nanoparticles, obesity-induced inflammation

## Abstract

Nanoscience is a multidisciplinary skill with elucidated nanoscale particles and their advantages in applications to various fields. Owing to their economical synthesis, biocompatible nature, and widespread biomedical and environmental applications, the green synthesis of metal nanoparticles using medicinal plants has become a potential research area in biomedical research and functional food formulations. *Gynostemma pentaphyllum* (GP) has been extensively used in traditional Chinese medicine to cure several diseases, including diabetes mellitus (DM). This is the first study in which we examined the efficacy of *G. pentaphyllum* gold nanoparticles (GP-AuNPs) against obesity and related inflammation. GP extract was used as a capping agent to reduce Au^2+^ to Au^0^ to form stable gold nanoparticles. The nanoparticles were characterized by using UV–VIS spectroscopy, and TEM images were used to analyze morphology. In contrast, the existence of the functional group was measured using FTIR, and size and shape were examined using XRD analysis. In vitro analysis on GP-AuNPs was nontoxic to RAW 264.7 cells and 3T3-L1 cells up to a specific concentration. It significantly decreased lipid accumulation in 3T3-L1 obese and reduced NO production in Raw 264.7 macrophage cells. The significant adipogenic genes PPARγ and CEPBα and a major pro-inflammatory cytokine TNF-α expression were quantified using RT-PCR. The GP-AuNPs decreased the face of these genes remarkably, revealing the antiadipogenic and anti-inflammatory activity of our synthesized GP-AuNPs. This study represents thorough research on the antiobesity effect of *Gynostemma pentaphyllum* gold nanoparticles synthesized using a green approach and the efficacy instead of related inflammatory responses.

## 1. Introduction

Obesity develops when energy intake is significantly higher than energy expenditure, resulting in fatty tissue expansion, characterized as hypertrophy and hyperplasia [1,2]. The obesity epidemic has increased dramatically due to lifestyle and dietary patterns [3]. Since 1975, the global obesity rate has tripled [4]. According to projections, 1.12 billion people will be obese, and 2.16 billion will be overweight by 2030 [5,6]. One of the most common health problems is associated with several metabolic disorders, including diabetes, hypertension, cardiovascular diseases, stroke, cancer, and nonalcoholic fatty liver diseases [7]. Adipose tissue secretes extreme glycerol, nonesterified fatty acids, proinflammatory cytokines (termed adipokines), and hormones including other factors [8]. Obesity can also be recognized as an inflammatory disease characterized by low-grade inflammation distinct from classical inflammation caused by infection [9]. Moreover, it was discovered more than a decade ago that inflammatory cytokines are highly expressed in obese rodents [10]. Multiple organs, including the pancreas, adipose, liver, skeletal muscle, brain, and heart, are involved in obesity-induced inflammation [11]. In addition to this, high-fat diet results in hyperlipidemia, followed by an increase in fatty acid oxidation, including reactive oxygen species (ROS) [12]. Furthermore, this activates the nuclear factor kappa beta (NF-κB) pathway and increases the secretion of the proinflammatory cytokines in various tissues [13].

Recent studies have found that immune cells, particularly monocytes/macrophages, are more active in obesity-induced inflammation and complications. In obese subjects, the number of an operational state of macrophages increases in adipose tissue, which significantly contributes to obesity-induced inflammation [14]. In 1993, Hotamisligil et al. reported that adipose tissue expressed a higher level of TNF-α cytokine in a rodent model of obesity [15]. Elevated levels of TNF-α are associated with the activation of multiple cell signaling pathways and increased lipolysis, inhibit the tyrosine kinase activity of the insulin receptor, and block insulin’s action. Moreover, the presence of high levels of lipopolysaccharide (LPS) in the bloodstream causes endotoxemia, which has been found in high-fat diet conditions [16]. The approved antiobesity drugs available nowadays are mainly effective in decreasing energy intake, but there are no approved drugs affecting energy expenditure [17]. Moreover, long-term use of antiobesity drugs can cause serious side effects [18]. Therefore, developing a novel drug to treat obesity and related metabolic disorders without side effects is inevitable.

Lately, nano-based therapies have become a leading platform for nanodrug delivery due to their upgraded progress in nanoscience and nanomaterials [19]. Nanoparticles are a prominent means of drug delivery in various metabolic diseases, including diabetes type 2 [20]. Recently, nanoparticles have been used to target specific tissues, including white adipose tissue (WAT) and brown adipose tissue (BAT), to remove the excessive lipids from adipocytes [21]. They can also reduce lipid levels, inflammation, and cancer [22]. Furthermore, this broad range of functions has unique characteristics of nanoparticles, such as enhanced solubility, stability, and bio-efficiency under metabolic conditions, including how they deliver drugs to the target. 

Gold nanoparticles (AuNPs) have become a considerable research area in the field of nanotechnology owing to their easy fabrication, stability, optical characteristics, oxidation resistance, and biocompatibility [23]. AuNPs have a wide range of applications in photodynamic therapy, X-ray imaging, drug delivery, sensing, and biomedicine [24]. AuNPs have been synthesized in most literatures by reducing gold ions with different reductases, such as citrate, hydrazine, and borohydride. 

Nevertheless, the green synthesis of metal nanoparticles using plants or biological organisms has gained more attention as it is nontoxic and environmentally friendly [25]. Over and above that, the fabrication of nanoparticles using plant extracts is a novel method to synthesize nanoparticles ideally, which are thought to form at neutral pH, ambient temperature, and low costs and in an environmentally benign manner [26]. Plants are “chemical factories” of nature, and they contain bioactive components, such as alkaloids, polyphenols, phenolic acids, proteins, sugars, and terpenoids. Those components possess functional groups that act as reducing agents to reduce the metallic ions and stabilize the nanoparticles [27].

*Gynostemma pentaphyllum* (GP), commonly known as “jiaogulan” in China, is a perennial herb from the Cucurbitaceae family [28]. *G. pentaphyllum* is traditionally used to combat elevated cholesterol levels, cough, and chronic bronchitis [29]. Particularly, the major active components of GP are gypenosides, which are structurally correlated with ginsenosides and have greatly attracted researchers’ attention [30]. Gypenosides have beneficial effects on inflammation, cancer [31], immune strengthening [32], antioxidant [33], hepatoprotection, and overweight [34]. In addition, jiaogulan contains other active compounds in different parts, including flavonoids, polysaccharides, sterols, and amino acids [35]. This plant is used to manufacture gold nanoparticles, which are different from bulk gold and broadly used in drug delivery. Besides, GP is available in Korea, and due to the accessibility of the plant source along with the medicinal value, we designed our study to synthesize gold nanoparticles from the plant extract. Additionally, the synthesis of gold nanoparticles has been conducted in an eco-friendly manner. Though the actual mechanism behind green synthesis is not elucidated yet, the green synthesis approach is the methodology for nanoparticle fabrication, the simplest and most effective time method. Nonetheless, we are reporting the first facile gold nanoparticle fabrication using *G. pentaphyllum* due to a higher amount of polyphenol, gypenoside III, and VIII and its application to combat obesity and related inflammation, which is the main defense system of the human body. 

## 2. Result and Discussion

### 2.1. Synthesis and Characterization of GP-AuNPs Using G. pentaphyllum Extract

Various plant extracts have been used in several works of literature to synthesize nanoparticles, including their wide range of applications [36,37]. This is the first study representing the biosynthesis of gold nanoparticles using the *G. pentaphyllum* (GP) plant, to the best of our knowledge. The synthesis of GP-AuNPs was confirmed by monitoring the color change of the reaction mixture. Before the incubation of the reaction mixture, the color was pale yellow. The reaction mixture turned to dark violet color after 10 to 20 min of incubation at 80 °C. In contrast, there was no color change in the control containing the same ratio of plant extracts. The deep violet color of the mixture unambiguously authenticates the formation of the gold nanoparticles by reducing aqueous HAuCl4 ions with GP extracts.

*G.pentaphyllum* contains many notable compounds similar to ginsenosides. According to previous reports, 25% of gypenosides are indistinguishable from ginsenosides. Peculiarly, gypenosides III, IV, VIII, and XII are strictly homogenous to ginsenosides Rb1, Rb3, Rd, and F2 [38]. The presence of various gypenosides and polysaccharides in GP has made this plant effective for the formation of nanoparticles. The green synthesis of zinc oxide nanoparticles using GP was reported earlier [39], but there is no report of the synthesis of GP-AuNPs. Our study proposed that an abundant number of phytochemicals in GP can act as reducing agents to reduce gold salts to synthesize gold nanoparticles. Besides, GP is available in Korea, and due to the accessibility of the plant source along with the medicinal value, we designed our study to synthesize gold nanoparticles from the plant extract. In addition, the synthesis of gold nanoparticles has been conducted in an eco-friendly manner. Though the actual mechanism behind green synthesis is not elucidated yet, the green synthesis approach is the methodology for nanoparticle fabrication, the simplest and most effective time method. Nonetheless, we are reporting the first facile gold nanoparticles fabrication using *G. pentaphyllum* and its application to combat obesity and related inflammation. 

**Figure 1:**
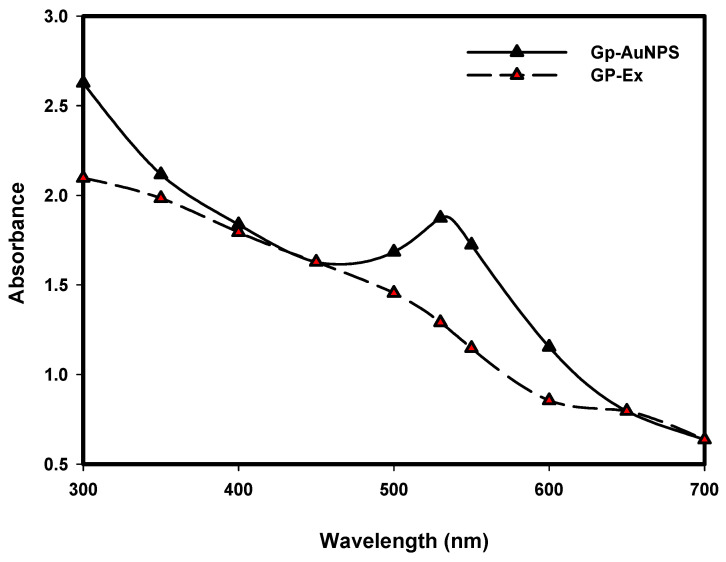
UV–VIS spectra of GP-AuNPs (black) and GP-Ex (red).

Ultraviolet-visible spectroscopy (UV–VIS) causes excitation by light at a particular wavelength with an individual peak at a specific wavelength called surface plasmonic resonance. The characterization of developed nanoparticles from GP extract was carried out by UV–VIS spectroscopy. According to studies, gold nanoparticles show the highest absorbance within 500–600 nm [40]. After scanning the reaction mixture within 300–700 nm, the maximum absorbance was observed at 532 nm, the identification peak for the gold nanoparticle (Figure 1). Even after storing the nanoparticles at 20 °C, there was no change in the UV–VIS spectra of the nanoparticle solution. 

**Figure 2:**
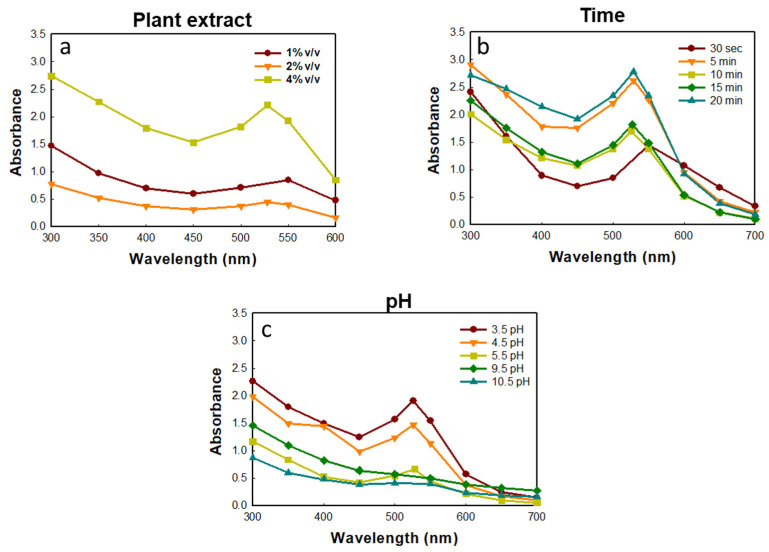
Optimization of GP-AuNP synthesis using different plant extract (**a**), time (**b**), pH (**c**).

The optimization of GP-AuNP synthesis was performed using different ratios (1%, 2%, 4%) of Gynostemma extracts. The results demonstrate that 4% of extracts have shown the maximum synthesis of GP-AuNPs shown in Figure 2a. This may be because a low concentration of extracts could not be enough to reduce Au+ to Au, which confirms the formation of NPs. The time required for the synthesis was optimized using various time intervals from 30 s to 20 min, and within that time, nanoparticle was synthesized at a higher amount (Figure 2b). In addition, 3.5 pH showed the highest absorption among all other pH’s tested (Figure 2c).

**Figure 3:**
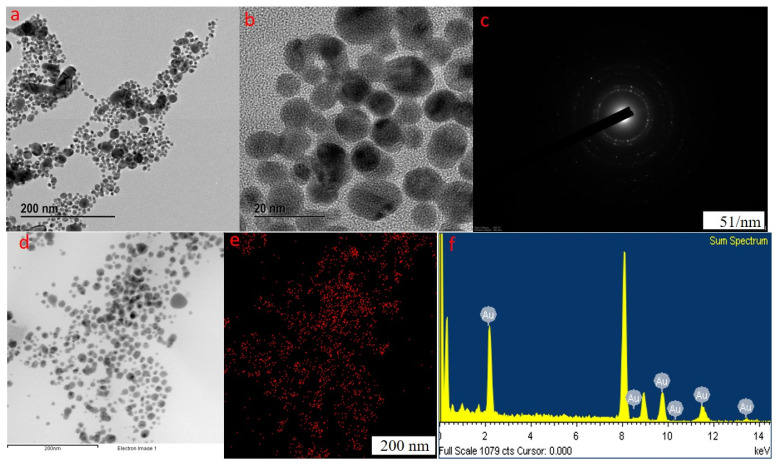
Field emission transmission characterization of GP-AuNPs on electron micrographs showed that GP-AuNPs are predominantly spherical in shape (**a**,**b**,**d**), and the selected area electron diffraction pattern revealed the crystalline nature of the biosynthesized nanoparticles (**c**). Elemental mapping and energy dispersive X-ray spectroscopy confirmed the purity of the nanoparticles (**e**,**f**).

The FE-TEM analysis results confirm the size and shape of the synthesized nanoparticles. In previous reports, hexagonal-, triangular-, and spherical-shaped gold nanoparticles were observed [41]. Our manufactured nanoparticles were spherical and measured in 20–200 nm (Figure 2a,b and Figure 3d). Furthermore, our synthesized nanoparticles were thoroughly monodispersed in nature, which is essential to eliminate polydispersity problems linked to biogenic synthesis. The selected area diffraction pattern (SAED) was utilized to examine the crystallinity of the GP-AuNPs (Figure 3c). The diffraction pattern in a ring form designates the crystalline nature of the formed nanoparticles. The maximum distribution of gold nanoparticles was inspected by elemental mapping, and results reflected in their respective electron micrograph image, which confirmed the predominant distribution of GP-AuNPs. In EDX analysis, the optical absorption band peak of GP-AuNPs exhibited at approximately 2.2 keV, resulting in the formation of pure nanoparticles (Figure 3e,f). Other metal ions were observed in the EDX spectrum belonging to the TEM grid used for the analysis. 

**Figure 4:**
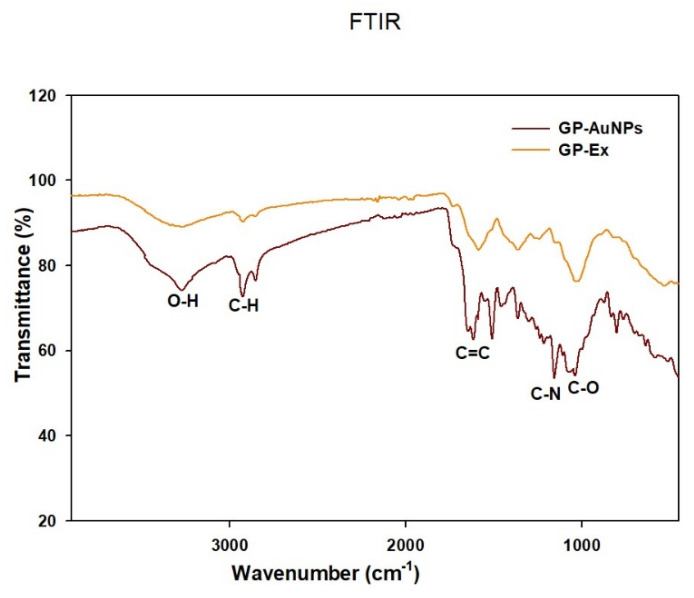
FTIR analysis of synthesized GP-AuNPs and GP-Ex.

The identification of organic compounds accountable for reducing and stabilizing metal ions into metal nanoparticles was confirmed using FTIR spectroscopy (Figure 4). The FT-IR analysis results exhibited the bonds at 3273.52, 2920.93, 1620.11, 1590.1, 1508.79, 1457.12, 1158.05, 1113.63, 1038.52, 803.38, 638.79, and 513.04 cm^−1^ for the biosynthesized GP-AuNPs. The 3500–3000 cm^−1^ bands are responsible for primary amine (N-H) and -OH stretching [42]. The peak at 3273.52 cm^−1^ specified the phenolic (-OH) in the GP-AuNPs, which appeared in GP extracts at 3278.39 cm^−1^. The absorption peak at 2920.93 cm^−1^ indicates C–H stretching comprising the entire band for alkane in polysaccharide and phenolic regions [43]. The peaks found from 1650 to 1450 cm^−1^ confirm the aromatic ring and c = c stretching. The bands between 1230 and 1020 cm^−1^ indicate the presence of the C–N group. Moreover, the absorption peaks between 1100 and 1010 cm^−1^ comprise C–O and C–H chemical bonds. [40]. According to FTIR analysis, aromatic organic compounds and protein in the GP extracts were responsible for the bioreduction of metal ions to metal nanoparticles. The appearance of proteins and amino acid residues is known to be accountable for the stability of biosynthesized nanoparticles. In addition, phenolic compounds of the GP extract can coat the nanoparticles and facilitate the stability of GP-AuNPs. Free amino groups and proteins are accountable for forming capping layers and preventing the aggregation of the nanoparticles. Over and above that, the active groups in the GP-AuNPs may bind some drugs or biomolecules owing to their free surface groups, which could play a crucial role in further drug delivery. 

**Figure 5:**
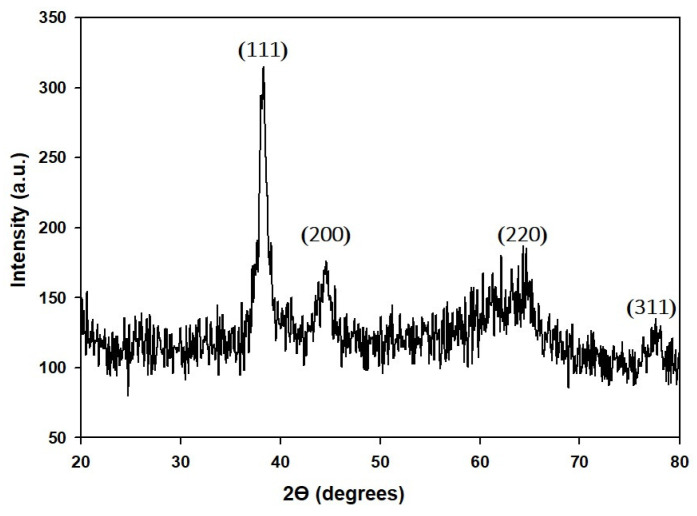
XRD analysis of GP-AuNPs.

The crystalline structure and purity of GP-AuNPs were observed by X-ray diffraction spectroscopy (XRD) patterns presented in (Figure 5). The diffraction peaks at 38.1°, 44.41°, 64.60°, and 78.31° were attributed to the (111), (200), (220), and (311) lattice planes, respectively. XRD result indicates that GP-AuNPs have a face-centered cubic (FCC) structure. This result is nearly close to gold nanoparticles’ XRD data published previously [44]. 

Therefore, the characterization studies authenticate the synthesized nanoparticles’ purity, stability, and crystallinity using the *G. pentaphyllum* plant.

### 2.2. Cytotoxicity Evaluation of Nanoparticles on 3T3-L1 and Raw 264.7 Cells

**Figure 6:**
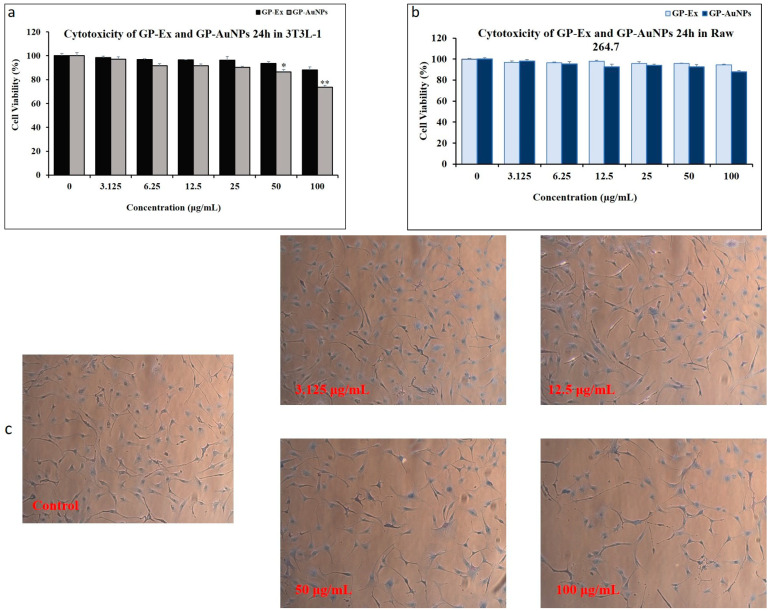
Cell viability of 3T3-L1 cell line on treatment with (**a**) GP extract and GP-AuNPs for 24 h. (**b**) GP extract and GP-AuNPs on Raw 264.7 cell viability for 24 h. (**c**) Trypan blue cell viability images before and after treatment with GP-AuNPs on 3T3-L1 cells. Each set of data represents the mean of triplicate experiment ± standard deviation. A significant difference between the groups was calculated using a two-tailed Student’s *t*-test. * < 0.05, ** *p* < 0.01 vs. control is used to represent a significant difference in cell viability of the sample compared with a nontreated control group.

The cell viability of GP-AuNPs was examined, following the incubation of 3T3-L1 and Raw 264.7 cells with various concentrations of GP extracts and GP-AuNPs for 24 h shown in Figure 6a–c. Cell survival rate was measured as a percentage of the viability of control or untreated cells. As displayed in Figure 6, after incubation with GP extracts and GP-AuNPs with concentrations ranging from 3.125 to 100 μg/mL, no significant toxicity or cell death was shown until 100 μg/mL in Raw 264.7 cells compared with the untreated cells. However, at 100 μg/mL in 3T3-L1 cells, GP-AuNPs started to show significant cytotoxicity. Therefore, we selected 3.125, 12.5, and 50 μg/mL for the subsequent experiments.

### 2.3. Effect of GP-AuNPs on Lipid Accumulation in 3T3-L1 Cells

**Figure 7:**
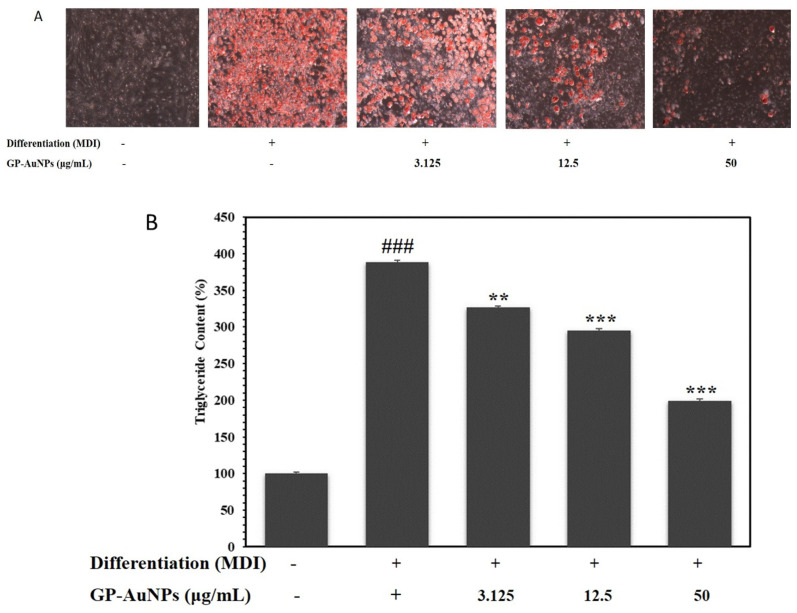
Inhibitory effect of the lipid accumulation for GP-AuNPs on MDI-induced 3T3-L1 adipocytes. (**A**) Fat droplets were measured by oil red O staining and observed using a microscope (at ×40). (**B**) The absorbance of lipid accumulation, which was oil red O dye, was dissolved in isopropyl alcohol (520 nm). The data are mean values of three experiments ± SEM; ### <0.001 compared with control, ** *p* < 0.01, *** *p* < 0.001 compared with the MDI.

**Figure 8:**
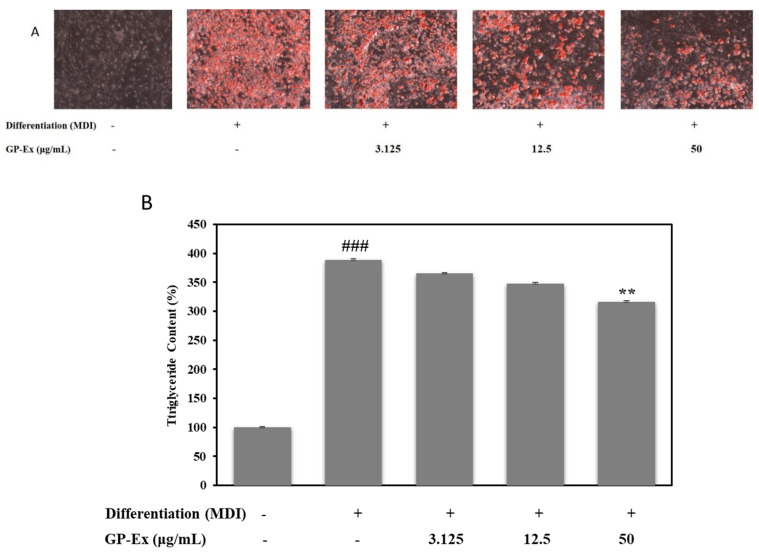
Inhibitory Effect of the lipid accumulation for GP extract on MDI-induced 3T3-L1 adipocytes. (**A**) Fat droplets were measured by oil red O staining and observed using a microscope (at ×40). (**B**) The absorbance of lipid accumulation, which was oil red O dye, was dissolved in isopropyl alcohol (520 nm). The data are mean values of three experiments ± SEM; ### <0.01 compared with control, ** *p* < 0.01 compared with the MDI.

Excess lipid accumulates in triglycerides due to insulin resistance, which causes an imbalance in energy homeostasis in adipocytes. To assess the antiobesity activity of our samples, the intracellular lipid droplets were stained with oil red O dye after differentiation of preadipocytes into mature adipocytes using an MDI medium with or without 3.125 to 50 µg/mL GP-Ex and GP-AuNPs simultaneously. The microscopic analysis showed a significant increase in lipid droplets in the cells treated with MDI medium (positive control). On the other hand, cells treated with GP-Ex and GP-AuNPs displayed lower lipid accumulation than the positive control (Figure 7 and Figure 8). The reduction of lipid accumulation occurred dose dependently. According to microscopic images, more than Gynostemma extracts showed that our synthesized nanoparticles using Gynostemma plants had decreased lipid droplets distinctly.

This result was further supported by quantitative analysis that came by the absorbance measurement. In Figure 7 a,b, the positive control groups showed a higher triglyceride content. In contrast, the cells treated with GP-Ex and GP-AuNPs had lower triglyceride content than the positive control. This study showed that GP-AuNP-treated cells showed lower triglyceride content than GP-Ex-treated cells. Cells treated with 50 µg/mL GP-AuNPs accumulated the lowest triglyceride. 

### 2.4. Assessment of Antioxidant Activity

**Figure 9:**
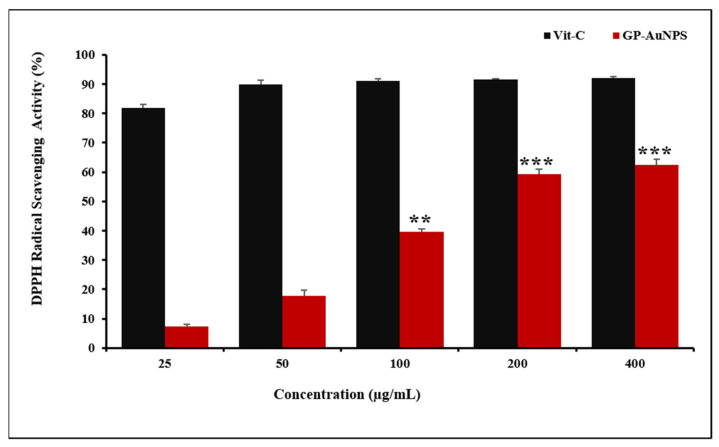
Dose-dependent in vitro DPPH radical scavenging and antioxidant activity of biosynthesized GP-AuNPs. Approximately 60% of DPPH inhibition was recorded at the 200 and 400 µg/mL. The data shown represent the mean values of three experiments ± SD. ** *p* < 0.01, *** *p* < 0.001 as compared with the Vit-C.2.5. Effect of GP-AuNPs on the LPS-induced NO production.

The level of reactive oxygen species elevates in the human body due to unfavorable conditions and causes cancer, aging, inflammation, and other disorders [45]. The free radical scavenging activity of GP-AuNPs was measured by DPPH assay. The DPPH reducing the ability of the AuNps was evaluated by the color change from purple to yellow at 517 nm. DPPH can accept electrons from antioxidants and reduce to DPPH2, resulting in color change. Figure 9 reveals the synthesized GP-AuNPs showing potential antioxidant properties. The scavenging activity of GP-AuNPs was increased dose-dependently. *Gynostemma pentaphyllum* is already proven to have antioxidant activity, and the possible reason for the budding antioxidant activity may be the presence of higher bioactive compounds [46]. The potential antioxidant capacity of GP-AuNPs can be ascribed to the adsorption of the bioactive molecules of GP by the NPs also, and spherical-shaped GP-AuNPs have a wider surface area. Moreover, the antioxidant ability of gold nanoparticles has been reported in many articles previously [40]. The recorded DPPH inhibition of our synthesized gold nanoparticles was approximately 60%.

**Figure 10:**
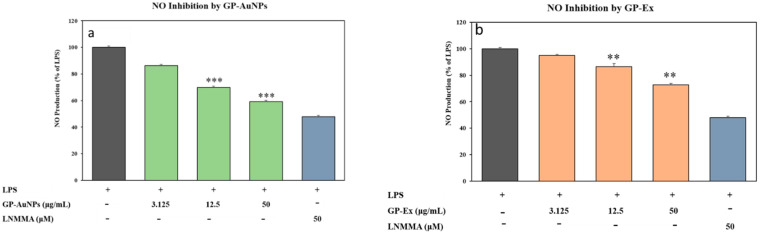
Effects on the production of NO GP-AuNPs (**a**) and GP extract (**b**). Macrophage RAW 264.7 cells were pretreated in both samples for 1 h and then stimulated with LPS (1 μg/mL) for 24 h. The concentrations of nitrite were measured as described in the materials and methods. The data shown represent the mean values of three experiments ± SD. ** *p* < 0.01, *** *p* < 0.001 as compared with the group treated with LPS.

Nitric oxide (NO) is the most dominant proinflammatory mediator that induces inflammation due to overproduction in abnormal situations, such as arthritis, inflammatory bowel diseases, and other inflammatory diseases of the respiratory system [47]. Consequently, subduing the overproduction of NO has become a potential prey in drug discovery for treating inflammatory disorders. We inspected the anti-inflammatory effect of GP-AuNPs on reducing NO production in LPS-induced RAW 264.7 cells. A common potent inhibitor of endothelial nitric oxide synthase, namely, L-NMMA, was used as a positive control in our study [48]. As shown in Figure 10, NO production is significantly higher in LPS-treated cells, whereas in both GP-Ex- and GP-AuNP-treated LPS-induced cells, NO production has decreased in a dose-dependent manner.

GP-AuNPs have shown more excellent anti-inflammatory activity than GP extract into the bargain. As we know, plant extracts have been used for years to synthesize nanoparticles due to their potential reduction and stabilization ability. Different plant extracts have different types and amounts of bioactive compounds. Ahn et al. [49] reported that gold nanoparticles synthesized from *Panax ginseng* extracts exerted significant anti-inflammatory properties in RAW 264.7 macrophages. As in this case, the nanoparticles are small in size, have a greater surface area, and can absorb the bioactive molecules of the plant extracts. These nanoparticles showed an increase in solubility, thus enhancing bioavailability. Hence, GP-AuNPs have shown better activity against obesity-induced inflammation than GP extracts.

### 2.5. Effect of GP-AuNPs on Reactive Oxygen Species in 3T3-L1 Preadipocyte Cells

According to a previous study, intracellular ROS production and lipid accumulation during adipogenesis are positively related [50]. Oxidative stress or intracellular ROS generation plays a pivotal role in increasing lipid accumulation and accelerating adipocyte differentiation [51]. On top of that, for managing the overproduction of proinflammatory cytokines, the expansion of adipose tissue through the adipogenesis process can lead to the formation of free radicals, which result in oxidative stress generation. This phenomenon highly contributes to the maturation of other metabolic disorders, including cancer [52].

**Figure 11:**
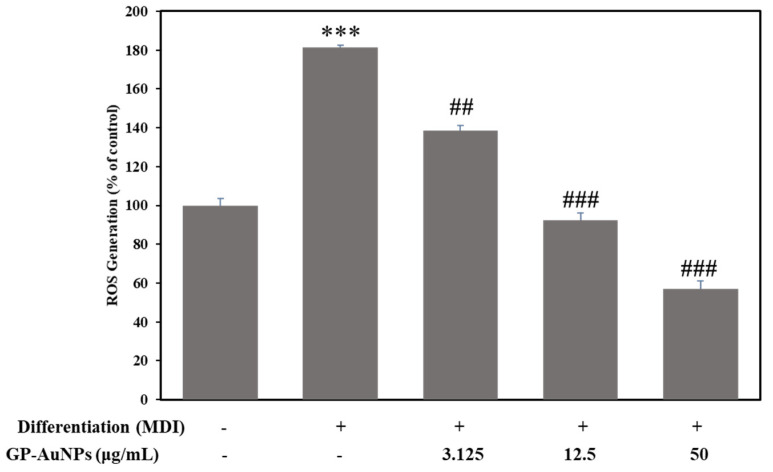
Inhibition of reactive oxygen species (ROS) generation by GP-AuNPs in MDI-induced 3T3-L1 adipocytes was determined by DCFDA method. GP-AuNP treatment reduced intracellular ROS production dose dependently. Data are expressed as a percentage of control. *** *p* < 0.001 MDI vs. control, while ## *p* < 0.01 and ### *p* < 0.001 GP-AuNPs vs. MDI.

Therefore, to examine cellular ROS generation in 3T3-L1 adipocyte cells, the suppressive effect of GP-AuNPs on ROS generation was quantified during adipogenesis using a DCFDA probe. According to our result, in comparison with untreated cells, intracellular ROS was notably increased in MDI-differentiated cells. Even so, when DMI-induced cells were treated with our sample GP-AuNPs, it dose-dependently decreased the ROS production (Figure 11). Consequently, due to having antioxidant properties, GP-AuNPs can inhibit ROS formation in 3T3-L1 cells.

### 2.6. Effect of GP-AuNPs on Gene Expression Levels during Adipogenesis and Inflammation

**Figure 12:**
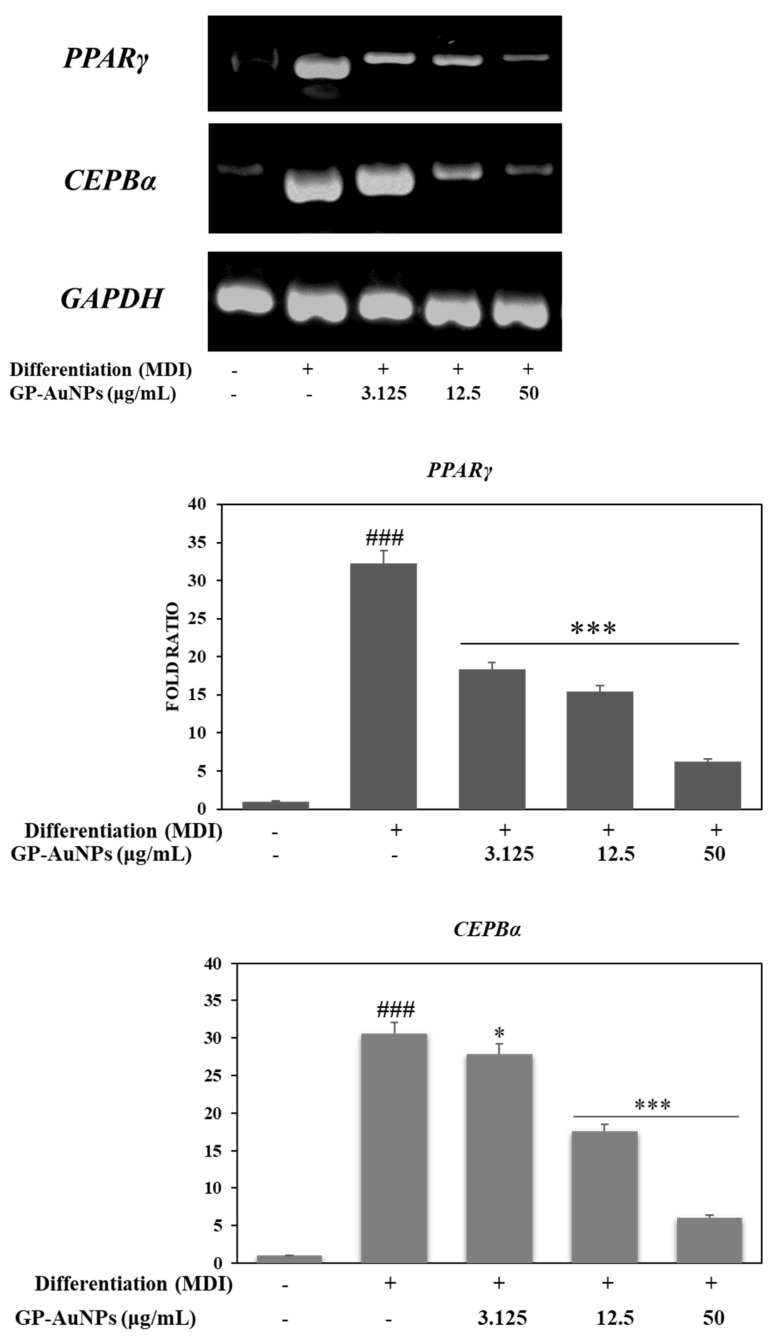
Effects of GP-AuNPs on mRNA expression levels of adipogenesis-related genes in 3T3-L1 cells. GP-AuNPs inhibit the RNA level of adipogenesis-related genes. ### <0.01 compared with control, * *p* < 0.05, *** *p* < 0.001 compared with the MDI.

The mechanisms of the antiobesity activities of GP-AuNPs on 3T3-L1 adipocyte differentiation and lipid accumulation were analyzed by RT-PCR. Both PPARγ and C/EBPα are critical markers for cellular responses related to adipocyte differentiation and lipid accumulation [53]. PPARγ is a master regulator, and the activity of PPARγ is thought to be mediated by the regulator C/EBPβ [54]. To investigate the inhibitory effect of GP-AuNP adipocyte differentiation, the expressions of PPARγ and C/EBPα were examined via RT-PCR. GP-AuNP treatment notably inhibited the PPARγ and C/EBPα expression in a concentration-dependent manner, as displayed in Figure 12. Our results suggest that GP-AuNPs inhibited adipogenesis by significantly downregulating the transcriptional factors.

**Figure 13:**
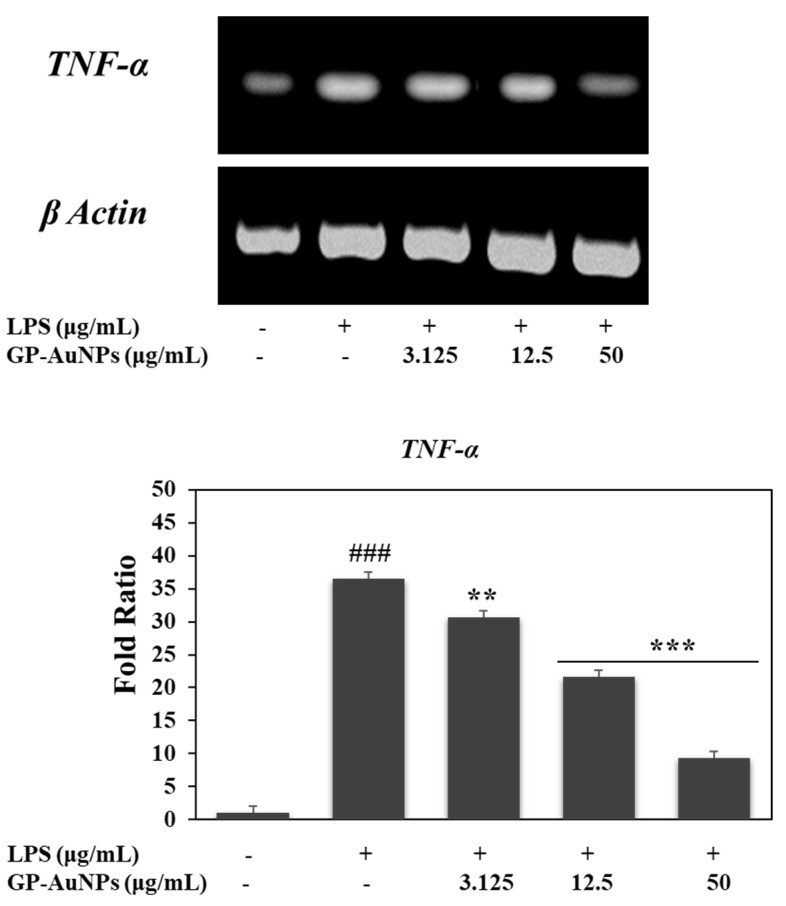
Effect of GP-AuNPs on the transcriptional activation of the inflammatory gene TNF-α in RAW 264.7 cells. RAW 264.7 cells were pretreated with the indicated concentrations GP-AuNPs for 1 h, and then stimulated with LPS (1 μg/mL) for 24 h. Subsequently, total RNAs were extracted, and the mRNA expression levels were determined by RT-PCR analysis and compared with those of β-actin. The data shown are representative of the mean values of three independent experiments ± SD. ** *p* < 0.01, *** *p* < 0.001 as compared with the group treated with LPS, and ### *p* < 0.001 as compared with the control.

To look into the anti-inflammatory effect of the GP-AuNPs, we measured the mRNA expression of TNF-α in LPS-induced RAW 264.7 cells in the presence or absence of GP-AuNPs. Many cytokines are responsible for the inflammatory response and tumor necrosis factor alpha (TNF-α), one of the most essential cytokines [55]. TNF-𝛼 levels are higher in the plasma and adipose tissue of obese individuals, and circulating levels were reduced with weight loss in humans [56]. We performed RT-PCR to determine the TNF-α gene expression at three concentrations of GP-AuNPs. Confirming our results, the GP-AuNPs have significantly inhibited the TNF-α expression (Figure 13).

## 3. Materials and Methods

### 3.1. Plant and Chemical

The plant sample (*Gynostemma pentaphyllum*) was obtained from Korea Hongsam Won CO., Korea. The 3T3-L1 mouse preadipocyte cell line was purchased from ATCC. The murine macrophage (RAW 264.7) cell line was collected from KCLB, Seoul, Korea. The preadipocyte cells were grown in DMEM (containing D-glucose, L-glutamine, sodium pyruvate, sodium bicarbonate) with 10% bovine calf serum (BCS) and 1% penicillin–streptomycin (P/S). DMEM and BCS were purchased from Welgene (Daegu, Korea), and P/S was purchased from GenDEPOT. Human recombinant insulin, dexamethasone, and 3-isobutyl-1-methylxanthine (IBMX) were obtained from Wako (Tokyo, Japan). Gold (III) chloride trihydrate (HAuCl4·3H2O) was purchased from Sigma-Aldrich Chemicals, USA.

### 3.2. Plant Extract Preparation

After collecting the plant samples, they were washed thoroughly using tap water and distilled water. Then the samples were shade-dried and grounded into powder form using a grinder. An amount of 100 mL distilled water was taken in a conical flask, and an amount of 5 g plant powder was added to the same flask. Then the solution was autoclaved at 100 °C for 40 min using an ultrasonicator to derive the contamination-free aqueous extract. Then the autoclaved extract was filtered through Whatman filter paper (No. 1). After that, the extract was centrifuged using a centrifuge machine at 5000 rpm for 15 min at ambient temperature to reduce unwanted compounds. Finally, we collected the supernatant and kept it at 4 °C for performing the following steps.

### 3.3. Synthesis of Gold Nanoparticles

The gold nanoparticles using *G. pentaphyllum* was prepared using the method described previously by Yi et al. [57]. In brief, 5 mL of plant extract was taken from the stock and mixed with 25 mL distilled water. Then 1 mM of HAuCl4·3H2O solution was added to the extract solution and kept at 80 °C for 1 h until the pale-yellow color of the extract was changed to purple color, which confirmed the formation of nanoparticles. These color changes indicate the reduction of ions to atoms (Au+ to Au). After the synthesis step, the nanoparticles were centrifuged at 15,000 rpm for 15 min, followed by thoroughly washing the particles with distilled water to remove waste materials. Finally, the gold nanoparticles (GP-AuNPs) were collected after air-drying overnight. For all further experiments, the nanoparticles were dissolved in PBS.

### 3.4. Characterization of GP-AuNPs

#### 3.4.1. UV–VIS Spectra Analysis

Gold nanoparticles can interact with a particular wavelength of light, leading to the unique optical properties of the gold nanoparticle. Therefore, the GP-AuNP formation was monitored by the absorbance spectra of the reaction mixture. The green synthesized nanoparticle solution was scanned in the range of 300–700 nm with a UV–VIS spectrophotometer (UV–VIS) (Ultrospec 2100 Pro, Amersham Biosciences, Amersham, UK) to confirm the bio-reduction of the gold salt.

#### 3.4.2. Field-Emission Transmission Electron Microscopy (FE-TEM) Analysis

The morphology, distribution, and purity of synthesized GP-AuNPs were decided by field-emission transmission electron microscopy (FE-TEM) with a 200 kV-operated JEM-2100F (JEOL) electron microscope. The partially purified nanoparticle solution drop was placed onto a carbon-coated copper grid. Subsequently, the samples were dried in a 60 °C oven and then transferred to an FE-TEM to analyze the nanoparticles’ configuration and size range. EDX analysis was performed to determine the purity of the nanoparticle, whereas elemental mapping was employed to investigate the distribution and location of the target component (Au). The electron diffraction pattern (SAED) was utilized to look into the crystalline structure of the synthesized nanoparticle [58,59].

#### 3.4.3. XRD Analysis

The X-ray diffraction (XRD) was carried out on an X-ray diffractometer using D8 Advance (Bruker), Germany, operated at 40 kV, 40 mA. CuKα radiation was used for scanning at a rate of 6°/min with a 0.02 step size, over the 2θ range of 20°–80°. The purified nanoparticles were collected after high-speed centrifugation and air-dried for the XRD sample preparation. Moreover, 5–6 mg of nanoparticle powder was submitted for XRD investigation [60].

#### 3.4.4. Fourier-Transform Infrared (FTIR) Spectroscopy Analysis

For FTIR investigation, dried nanoparticle powder was scanned over 4000–450 cm^−1^ at a resolution of 4 cm^−1^ using a PerkinElmer Spectrum One FTIR spectrometer. FTIR was performed to confirm the interaction between the functional groups available on the nanoparticle as catalytic and capping agents. The recorded spectra were plotted as % of transmittance versus wavenumber (cm^−1^) [61].

#### 3.4.5. Stability of A-AuNPs

GP-AuNP stability was examined by UV–VIS performed at different time intervals and pH at ambient temperature. To check the time stability, the absorbance of the collected nanoparticle was determined from 30 s to 20 min. Similarly, the pH stability was selected in the range of 3–10 pH with a UV–VIS spectrophotometer [62].

### 3.5. Cell Culture

For cell culture, 3T3-L1 fibroblast preadipocytes were grown in complete media containing DMEM with 10% BCS and 1% P/S and incubated in a 5% CO_2_ incubator. RAW 264.7 cells were cultured in DMEM medium supplemented with 10% fetal bovine serum (FBS) and 1% penicillin/streptomycin under the same conditions. Preadipocytes were differentiated from matured adipocytes. Preadipocytes were plated in a 12-well plates at a density of 2 × 105 cells/well. Cells were kept to reach 90% confluence, and after 48 h, cells were stimulated using a differentiation medium or adipogenesis-inducing medium (MDI). MDI was prepared using 1 µM dexamethasone, 0.5 mM IBMX, and 10 μg/mL insulin to complete the medium. On day 2, cells were stimulated with adipogenesis maturation medium (10 μg/mL insulin to complete the medium) with or without the addition of GP-AuNPs, and further incubated for two 5–8 additional days. On day 8, the preadipocytes were differentiated to matured adipocyte cells and displayed lipid droplets when viewed under a microscope. Cells that were cultured only on a complete medium were used as a control.

### 3.6. Cytotoxicity Assay

For cytotoxicity assay, 3T3-L1 preadipocyte cells and RAW 264.7 cells were seeded at a density of 1 × 104 cells/well in 96-well plates and allowed to attach overnight at 37 °C in a 5% CO_2_ incubator.

Additionally, 3T3-L1 preadipocytes were seeded in 96-well plates (2 × 104 cells/well) and allowed to attach overnight in DMEM/high glucose. After discarding the medium, various GP and GP-AuNP (3.125 to 100 µg/mL) concentrations were added to each well. The cells were then incubated for 24, and untreated cells were used as controls. After 24 h, the medium was replaced and MTT (3-[4,5-dimethylthiazol-2-yl]-2,5-diphenyltetrazolium bromide) solution was added (20 µL/well) and incubated for 2 to 3 h. Finally, 100 µL DMSO was used to stain the cells to produce the formazan crystal into a colored solution. The absorbance was measured at 570 nm using a microplate reader (BioTek Instruments, Inc., Winooski, VT, USA).

### 3.7. Lipid Accumulation and Triglyceride Measurement Assay

On 14th day, an oil red O assay was performed to visualize lipid droplets that confirm adipogenesis in 3T3-L1. Then, the matured adipocytes were washed with 1 × PBS, followed by the fixation using 10% formalin for 1 h or a few days. After the fixation, cells were soaked in 60% isopropanol and left to dry completely. Matured adipocytes were then stained using oil red O solution for 30 min and washed with distilled water to remove the excess stain. An inverted light microscope captured phenotypic changes in fully differentiated cells (Nikon Instruments, Melville, NJ, USA). Finally, 100% isopropanol was added to the mature adipocytes to measure the triglyceride content (lipid accumulation) of adipocytes. Then the reactions were incubated at room temperature for 10 min, and the absorbance was read at 630 nm [63].

### 3.8. RNA Isolation and RT-PCR

According to the manufacturer’s recommendation, total RNA was isolated from GP-AuNP-sample-treated adipocyte cells and Raw 264.7 cells with TriZol LS reagents (Invitrogen, Carlsbad, CA, USA). cDNA was synthesized from 1 µg of total RNA using a commercial cDNA synthesis kit (OneBio, Lithuania, EU) following the recommended protocol. The cDNA synthesis condition was 42 °C for 1 h and then 72 °C for 5 min. Then the synthesized cDNA was used for amplification of the targeted gene. The primers used for the RT-PCR are as follows:
**Genes****Forward Primers****Reverse Primers**PPARγATGGGTGAAACTCTGGGAGATTAGCTTCAATCGGATGGTTCTTC/EBPαAGGTGCTGGAGTTGACCAGTCAGCCTAGAGATCCAGCGACTNF-αAGGGGAAATGAGA GACGCAATTCCCCATCTCTTGCCACATGAPDH β-actinGTATGACTCCACTCACGGCAAA AGCCATGTACGTAGCCATCCGGTGTGGCTCCTGGAAGATG TCCCTCTCAGCTGTGGTGGTGAA

The reverse transcription-polymerase chain reaction (RT-PCR) was performed for PPARγ and CEPBα at 94 °C denaturation temperature for 30 s, the response was annealed at 54 °C, and extension was performed at 72 °C for 1 min and 30 cycles. For TNF-α, the reaction was denatured at 94 and 54 °C annealing temperatures using 32 cycles. To allow reannealing of the amplified product, the temperature was held at 72 °C for 5 min after completing the final cycle. Finally, the electrophoresis and PCR samples were loaded in 1% agarose gel.

### 3.9. Free Radical Scavenging Activity of GP-AuNPs

The antioxidant properties of GP-AuNPs were found by DPPH analysis with the slight modification of the previous method explained by Abbai et al. [64]. Different concentrations of GP-AuNPs (25, 50, 100, 200, 400 μg/mL) were selected, and 20 μL of nanoparticles solution was added to 180 μL of 1 mM methanolic DPPH solution. Then the solution was incubated in the dark for 30 min. After the dark incubation, absorbance was quantified at 517 nm using vitamin C as a positive control. The formula determined the free radical scavenging activity, % inhibition = ([control OD − sample OD]/control OD) × 100. Three replications were performed to enhance the reliability of the analysis. The radical scavenging activity was determined as % inhibition: ([control OD − sample OD]/control OD) × 100. To enhance the reliability of the analysis, three replications were performed.

### 3.10. Measurement of Cellular ROS

The 2′,7′-dichlorofluorescein diacetate (H2DCFDA) is a cell-permeable fluorogenic probe commonly used to determine the level of reactive oxygen species (ROS) generation. The intracellular ROS production was assessed by washing the differentiated cells with PBS at room temperature. After washing the cells, a nonfluorescent probe of 10 μM H2DCFDA (Sigma, St. Louis, MO, USA) was added for 30 min and incubated in the dark at 37 °C. Finally, the fluorescence emission intensity was determined between 485 and 495 nm, respectively, with a Spectra Fluor multiwell fluorescence reader (Tecan, Maninder, Austria) according to a previous protocol by Simu et al. with minor modification [65].

### 3.11. Measurement of Nitrite Levels

RAW 264.7 cells were pretreated with various concentrations of GP and GP-AuNPs for 1 h. Then the cells were stimulated with 1 μg/mL LPS, followed by incubation for 24 h. For the quantification of the nitrite level in the medium, the Griess reagent was used. In a nutshell, 100 μL of the Griess reagent was mixed with 100 μL of supernatant. Eventually, the absorbance was measured at 540 nm using a microplate reader (BioTek Instruments, Inc., Winooski, VT, USA) [66].

### 3.12. Statistical Analysis

Data are expressed as means ± SEM of three independent experiments. The mean values of the treatment groups were compared with untreated groups using Student’s *t*-test. Statistical significance was accepted at a level of *p* < 0.05.

## 4. Conclusions

Recent studies focus more on obesity and obesity-related metabolic disorders, including inflammation due to increased amount of proinflammatory cytokines into the circulation and the reduction of anti-inflammatory adipokines in obese conditions that lead to local and systemic inflammation. Therefore, to invent a possible therapeutic area to combat obesity and obesity-mediated inflammation, we investigated the effect of *G. pentaphyllum* gold nanoparticles against obesity and obesity-mediated inflammation. Moreover, the gold nanoparticles were synthesized using a green approach, which is a simple, efficient, and nontoxic method.

To assess antiobesity activity, we examined the effect of GP-AuNPs on intracellular lipid accumulation. Our sample significantly decreases lipid accumulation and suppresses adipogenesis by inhibiting the significant adipogenesis genes, such as PPARγ and C/EBPα. Moreover, adipocyte may change their phenotype from white adipose tissue (WAT) to brown adipose tissue (BAT), which is known as browning. Brown adipose tissue (BAT) utilizes energy as heat through the oxidation of fatty acids and plays an important role to combat obesity. After treating, our synthesized nanoparticle adipocytes may change their phenotype and increase energy utilization and facilitate antiobesity activity. In a future study, we can focus on the browning capacity of GP-AuNPs. We also measured the obesity-related anti-inflammatory effect of GP-AuNPs. We quantified the NO level, which is a major signaling molecule in inflammation, and NO production reduced significantly with GP-AuNPs. Our sample remarkably decreased the NO production. To be more specific, we measured the gene expression of a major proinflammatory cytokine known as TNF-α to assess its anti-inflammatory effects. GP-AuNPs also suppressed this cytokine notably. Then again, in an obese state, oxidative stress is increased due to low antioxidant activity along with increased proinflammatory cytokines. We examined the reduction of increased ROS during obesity. Our GP-AuNPs significantly suppressed the intracellular ROS production in 3T3-L1 cells. In sum, our synthesized GP-AuNPs appreciably diminished obesity and obesity-related inflammation. This supports the assumption of other literature reported on the mechanism of obesity and related inflammation.

## Data Availability

We do not wish to make the data publicly available as further research is being undertaken based on this study.
